# Crisis Preparedness and Systemic Risk: The Role of Municipal Leaders in Disaster Risk Governance in Norway

**DOI:** 10.1111/sjop.70015

**Published:** 2025-08-23

**Authors:** Sofie Steinsund, Ilan Kelman, Gianluca Pescaroli, Jarle Eid

**Affiliations:** ^1^ SLATE (Centre for the Science of Learning & Technology) University of Bergen Bergen Norway; ^2^ Department of Risk & Disaster Reduction University College London London UK; ^3^ Institute for Global Health University College London London UK; ^4^ UiT The Arctic University of Norway Tromsø Norway; ^5^ Cambridge Centre for Risk Studies Cambridge University Cambridge UK; ^6^ Department of Psychosocial Science University of Bergen Bergen Norway

**Keywords:** crisis leadership, crisis preparedness, disaster, municipality, resilient society, training, trust

## Abstract

This study explores how Norwegian leaders in rural municipalities attend to disaster risk governance and prepare for resilient response to threats from systemic, interconnected, and cascading hazards. Systemic risk management in the Norwegian municipalities will depend on the context and how critical organizational processes are managed by the municipal leaders. Following the COVID‐19 pandemic and an increased awareness of climate‐related threats, 12 Norwegian municipal leaders (7 men and 5 women) participated in semi‐structured interviews on crisis management and disaster risk preparedness in their municipality. The analysis identified three main themes: (a) *Facing the unknown:* addressing their emotional perception of risk, responsibilities, and the constant struggle to balance their everyday tasks and prioritize crisis preparedness; (b) *Procedures are needed but relations are key:* pointing to the need for collaboration, the value of trust, and to maintain good interpersonal relations; (c) *We need to train:* acknowledging the value of training and crisis preparedness in searching for viable ways to prepare for the unexpected. The study underscores the role of municipal leaders and highlights the value of interpersonal relations and trust when faced with decision dilemmas, uncertainty, and training needs in local disaster risk governance.

**Summary**

- This study explores how leaders in rural Norwegian municipalities struggle with balancing everyday responsibilities and crisis preparedness, when dealing with unknown and complex risks.
- While procedures are important, the study highlights that interpersonal relationships, collaboration, and trust are key to effective disaster risk governance.
- Leaders recognize the importance of regular training and realistic exercises to build resilience, trust and interpersonal relations to face unexpected events.

## 1 Introduction

Hazards and vulnerabilities come in many forms. The interdependence and complexity of modern society present an increased risk to continuity management (e.g., to quickly restore vital operations in the event of disruption). Effective leadership is essential for a resilient response and requires municipal leaders to overcome barriers between sectors, regulatory levels, and professions (Almklov, Antonsen, Bye, and Øren 2018; Almklov, Antonsen, Størkersen, and Roe 2018; Nilsson 2010). A core aspect of disaster risk governance is to ensure that safety systems are flexible, responsive, and resilient to changes and unexpected events. Despite the presence of legislation and policy guidelines in support of continuity management, crisis preparedness may be ineffective, and resources may be inadequate (Enander et al. 2015; Hede 2017). Municipalities and boroughs play a crucial role in ensuring the continuity of basic services and securing safe livelihoods for their inhabitants (Olsen et al. 2007). When faced with risk and hazards such as the recent COVID‐19 pandemic (Lien et al. 2023; Steinsund and Eid 2023), the threat of natural hazards (DSB 2023), war, or a combination of these, municipalities are supposed to mobilize a resilient response to ensure the continuity of basic services for their inhabitants.

In rural municipalities, disaster risk governance frequently relies on dedicated individuals with personal crisis experience (Øren et al. 2016). Municipal leaders are faced with the dilemma of allocating public resources to prepare for low‐probability hazards versus addressing everyday community needs even though these priorities are complementary and mutually supportive in support of promoting local health and livelihood (Blaikie et al. 2014; Olsen et al. 2007).

This study explores how leaders in rural municipalities in Norway approach disaster risk governance and prepare for resilient responses to systemic, interconnected, and cascading hazards (Pescaroli and Alexander 2018; Suppasri et al. 2021). We will first provide an overview of current research on disaster risk reduction (DRR), systemic risk, and vulnerability as they inform disaster risk governance in municipalities. Then, we will explain how the context and operational role of Norwegian municipal leaders in emergencies inform our research questions.

## 2 Literature Review

Municipalities play a vital role in addressing hazards and vulnerabilities despite significant differences in resources, plans, and preparations. Perspectives often vary even within the same local authority (Nilsson 2010). When resources need to be mobilized from within or outside, it is essential to make informed decisions about what actions to take and who will undertake them. Several studies have emphasized the salience of a community‐based approach in support of disaster risk governance and resilience in small municipalities. A study from Pugad Island, Philippines, emphasized the importance of leadership practices, human rights values, and the inclusion of vulnerable groups in disaster risk reduction DRR plans to maintain confidence in and support of local governance (Abenir et al. 2022). In Italy, resilience factors for managing severe floods included financial resources and benign external relations, while bureaucratic constraints and poor urban planning posed challenges (Sciulli et al. 2015). In 2017, a rockfall‐induced tsunami in Greenland highlighted the role of a collectivistic culture, local knowledge, and trust in a resilient response to infrastructure relocation and emergency preparedness (Taarup‐Esbensen 2022). These brief examples emphasize the need to consider contextual and interpersonal relations when assessing DRR in rural areas.

Existing research often misses the dual role of decision makers in conducting business as usual while also preparing for potentially disruptive and adverse scenarios. Municipal leaders and local authorities are therefore frequently challenged to identify new strategies and tools that can allow them to operate with more flexibility in situations with reduced capacity (Pescaroli et al. 2023). In addition to developing contingency plans, municipal leaders must be prepared to serve in the crisis management committee in their municipality (Hede 2017). A looming crisis will require municipal leaders to leave their day‐to‐day routines and respond to the unfolding emergency. They are expected to take center stage in the immediate and long‐term crisis response and attend to logistical as well as emotional needs (Eid, Hansen, et al. 2023). This role transition is challenging and will in part depend on their ability to generate trust and mobilize collective action to overcome the crisis (Hyllengren et al. 2011).

The COVID‐19 pandemic reminded municipal leaders that risk is systemic, interconnected, and cascading (Suppasri et al. 2021). Overnight, municipality leaders had to shift their attention from daily routines to crisis management. In Norway, the government introduced significant infection control measures that had disruptive effects on the educational system (Lien et al. 2023; Steinsund and Eid 2023) and the business community (Dale Oen et al. 2022). The pandemic revealed how concurrent risks and cascading effects from the healthcare sector could have disruptive consequences to the local municipalities (Pescaroli et al. 2021, 2023). The literature on cascading effects of crises requires more work and in‐depth studies of how rural municipalities manage cascading situations.

### 2.1 Crisis Preparedness and Resilience Training

Risk and vulnerability analysis can be seen as an organizational learning process, where the goal is to facilitate structural changes in the organization (Wang and Ahmed 2003). Still, an inquiry into 15 Swedish municipalities revealed that the risk and vulnerability analysis did not serve as a vehicle for organizational learning and crisis preparedness (Eriksson 2023). In Norway, economic and political systems present limitations to the local crisis preparedness work (Orderud and Kelman 2011), pointing to a need to understand risks and preparedness in a wider context (Kelman and Rauken 2012).

Responding to crises requires the ability to adapt to unknown moral, physical, or mental stressors (Ketelaars et al. 2024). To this end, the training of local crisis management teams needs to raise awareness of risk assessment and disaster risk governance. In addressing this need, Eid, Hansen, et al. (2023) proposed a conceptual roadmap in support of crisis management training. They suggest that future team training designs should showcase how contextual factors, technology, and team interaction would influence complex problem solving in the municipality. A promising approach to this end could be in the form of virtual exercises where participants interact to provide a deeper understanding of roles, responsibilities, and capacities to mobilize a resilient response. Such virtual or distributed team training became widely accepted during the pandemic (Eid, Brattebø, et al. 2023) and could present a feasible solution to crisis management training for municipality leaders. Distributed crisis response training would also address the interconnectedness among critical infrastructure and how inter‐agency training could facilitate collective coordination and improvisation (Frykmer et al. 2018). Because crisis situations are complex and can be difficult to manage, training design needs to be carefully developed and evaluated. Heino et al. (2021) suggest that a new mindset is needed to prepare emergency response actors for the complex, versatile, and dynamic situations they may face. A recent comparative study of the local fire and rescue services in Bergen, Norway, and boroughs in London, UK, during the COVID‐19 pandemic underscores this point. Four common themes emerged related to emotional experiences, how to maintain readiness, continuing to serve the community, and professionalism and learning (Eid et al. 2025). These themes highlight the need for flexibility in the planning of local crisis response, rather than comprehensive plans, and the need to encourage local leadership that understands emergency responders' requirements. Thus, municipality leaders serve an important role in support of individual and organizational resilience, and the interface between them.

### 2.2 The Current Study

The study will contribute to providing new empirical observations on disaster preparedness and how municipal leaders in Norway approach disaster risk governance. Although there is more research on the emergency services, less research has focused on the role of municipality leaders. In the following, we will explore these research questions:

– How do Norwegian municipal leaders prepare for low‐probability, high‐impact hazards?
– What do they consider to be the most important aspects of disaster risk governance in their municipality?
– How could local crisis management training be further developed in the municipalities?

## 3 Method

### 3.1 The Study Context

Norway has 356 municipalities and the crisis management structure spans three administrative levels (i.e., local, regional, and national) of governance. A comprehensive set of regulations, guidelines, and audit mechanisms is established to oversee the system (DSB 2018). Numerous significant threats exist across Norway (NOU 2023:17 2023). For instance, in 2022, about 57% of the Norwegian municipalities had been exposed to extreme weather incidents such as snow, landslides, or floods (DSB 2023).

The Norwegian crisis management system is based on four core principles (DSB 2023); (a) *proximity* (i.e., that an unwanted incident must be handled at the lowest possible level); (b) *similarity* (i.e., the crisis response organization should be as similar as possible to the ordinary organization); (c) *responsibility* (i.e., the department or unit that has the daily responsibility for a specific domain, will also be responsible for social security in that domain); and (d) *cooperation* (i.e., all social security actors have an independent responsibility to ensure the best possible cooperation with relevant actors and businesses).

Although municipalities are responsible for the strategic and long‐term aspects of crisis management, the emergency services handle the immediate, tactical responses (Antonsen and Ellingsen 2019). The municipality leaders and the local crisis response board will in most cases have a supportive role, for example, managing evacuation, securing safe shelter and food, alerting and informing the public, and providing support to first responders (DSB 2018). Although both services are essential for a comprehensive crisis response, relatively few studies have provided empirical observations on disaster risk preparedness in municipality managers. The municipality managers are first to realize the apparent mismatch between the public expectations of what the municipalities are expected to do and their capacity and training (Øren et al. 2016). Survivors from disasters expect the authorities to help them in a fair, compassionate, equal, and reliable manner and to aid in fulfilling event‐related practical needs (Jong and Dückers 2019). At the same time, most municipality managers and elected political leaders have little if any formal education and training in emergency management, in contrast to the emergency services who are highly specialized and trained. Less is known about how the municipal leaders assess their proficiency, resilience, and crisis preparedness.

### 3.2 Research Design

An exploratory qualitative research design was considered suitable to obtain rich descriptions of how the municipal leaders assess, perceive, and experience risk and crisis preparedness in their municipalities (Klenke et al. 2016; Patton 2015). A semi‐structured interview guide was developed with the aim to approach the active duty, executive management (i.e., municipal director, senior leaders) to get a firsthand understanding of how municipal leaders perceive systemic risk and crisis preparedness in the municipality. The municipal director serves a key role in chairing the municipality crisis committee in close collaboration with first responders, regional and local resources. Politically elected leaders such as the mayor, deputy mayor, or city council representatives will mostly serve in supporting roles and were therefore not included in the present study. In the analytical process, our preconceptions were adjusted in line with the empirical data until a new agreed‐upon understanding of the phenomenon was achieved (Malterud 2001, 2012).

### 3.3 Participants

The sample in this study consisted of 12 municipal leaders (ML) in senior administrative roles (7 men, 5 women) from 11 different Norwegian municipalities, primarily located in the western part of Norway. Six of the municipalities were categorized as small (< 4999), two were middle‐sized (5000–19,999), and three were large (> 20,000 inhabitants), according to a common classification of Norwegian municipalities reported in Table 1 (Kringlebotten and Langørgen 2020).

**TABLE 1:** Classification of the 11 municipalities in the study, according to population size.

Municipality categorization	Population	Number of municipalities represented
Small	0–4999	6
Middle	5000–19,999	2
Large	20,000<	3

*Note:* Two of the 12 informants belonged to the same municipality.

All the municipality leaders had experience from recent climate‐related incidents and the COVID‐19 pandemic. The participants were recruited using a purposeful sampling approach (Klenke et al. 2016, s. 9) to ensure characteristics such as leader position and experience, as well as variation in municipality size. The participants were approached by an initial e‐mail invitation where they gave informed consent and agreed on a suitable interview time. Eight of the leaders held municipal director positions; the others were assistant municipal directors, municipal managers, or had experience with leading the local psychosocial response team. All leaders had extensive experience as municipal leaders, ranging from 6 to 24 years (13 years on average).

### 3.4 Data Collection

**FIGURE 1:**
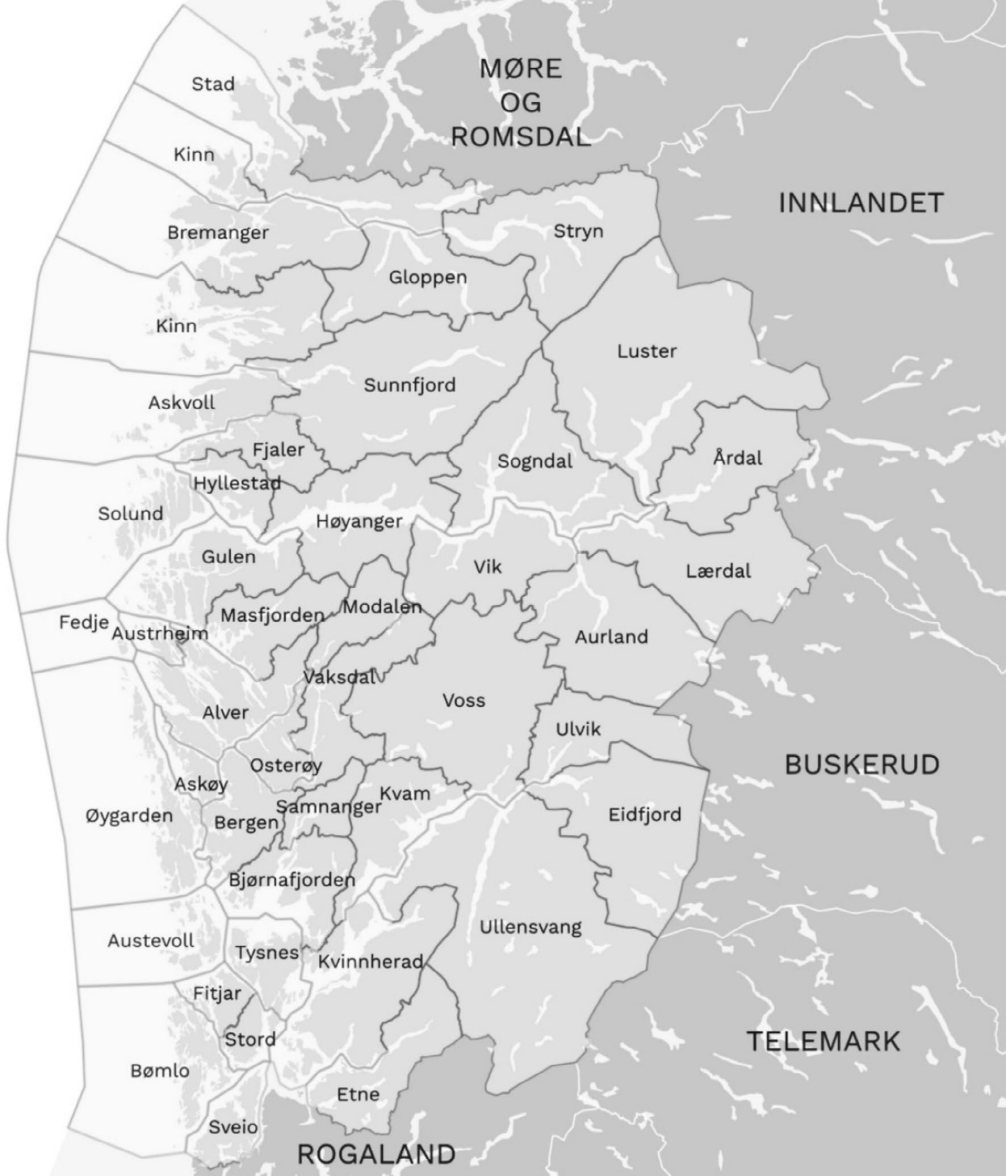
Overview of municipalities in Vestland county.

All interviews were conducted in Norwegian by the first author over a 4‐month period from December 2022 to March 2023. The interviews were conducted online using Microsoft Teams. This provided a flexible solution to establish a mutual video and audio dialog with busy municipal executives, regardless of their geographical location. The participants were informed that only speech was recorded on a separate device. The interviews lasted between 30 and 45 min. Immediately after the interviews, the audio files were transcribed. To protect the identity of the participants, no names or background data were transcribed, and a separate code was ascribed to each participant. As the data collection progressed, the researchers frequently reviewed the information from the interviews to assure consistency and to understand when the threshold of data saturation was reached. After completing 12 interviews, we observed that new data started to repeat what had already been expressed in previous interviews, and we concluded that a sample size of 12 participants would provide sufficient variation to explore the research questions and main goal of the study (Malterud 2012). The digital recordings were deleted after transcription, keeping only the anonymized transcripts for the analysis (Figure 1).

The interviews followed a semi‐structured format (Kvale and Brinkmann 2015, 46–49), with examples of themes, questions, and probes reported in Table 2. An introductory question about their experiences from crisis situations in the municipality set the stage for further probes into crisis situations and crisis management. The interview progressed by exploring their experiences from teamwork and collaboration during crises, asking about factors that were important in support of local preparedness and resilience in the municipality. Another theme focused on their experience from crisis management training and their opinions on how to improve future crisis preparedness in the municipality. Probing questions were used to elicit richer, deeper, and detailed information, providing additional insight into important aspects of crisis leadership and preparedness at a deeper level (Robinson 2023).

**TABLE 2:** Examples of interview themes, sample questions, and probes.

Themes	Sample question	Probes
Experience with crises	*If you think about one specific crisis you've experienced or might experience—what could it be?*	*Can you tell me a bit more about this? About the sequence of events?* *What was/would be your role in this situation?*
Teamwork and collaboration during crises	*What do you think would be important for good team collaboration in a crisis?*	*What will be important to you as a leader? What do you need from those around you?* *What challenges have you experienced/do you foresee?*
Perception of municipal preparedness and resilience	*How well equipped is your municipality/agency to respond to various critical societal incidents?*	*Could you give examples of situations you are well prepared for and situations that you are not prepared for?*
Future local preparedness	*What do you think can (should) be done to strengthen preparedness in your municipality?*	*What do you see as the biggest obstacles to better public safety in the municipality?* *What is working well?*
Education and training for crisis preparedness	*What is your experience with crisis training and education in your municipality/agency?*	*What do you think is working well when it comes to training?* *What could be improved?*

### 3.5 Data Analysis

Systematic text condensation (STC: Malterud 2012) was used as a strategy to analyze the transcribed data. This strategy is a pragmatic approach for thematic cross‐sectional analysis of qualitative data (Malterud 2001). The analysis was conducted by the first author, in close collaboration with the last author, according to the four steps in STC (Malterud 2012). The first step consisted of reading the transcribed material in its entirety to obtain an overall impression of the data. Here, preliminary themes were identified.

In the next step, a thorough reading of the transcripts was carried out to identify and develop meaning units related to the study question. In this coding process, related meaning units were classified and sorted into code groups based on the preliminary themes from step one.

In the third step, each code group was treated as an analytical unit. The content of the meaning units was condensed and clustered, and sorted into subgroups, grasping the diversity of nuances within each code group. Then, each subgroup was characterized by a condensate, an artificial quote that summarizes and represents the phenomenon that the subgroup describes (Malterud 2012). Table 3 shows groups and subgroups with examples of meaning units connected to each group. In this process, the code names and distinction between groups were adjusted in accordance with a broadened understanding of the phenomenon.

**TABLE 3:** Overview of the analytical process in the study.

Step1: Overall impression	Step 2: Meaning units		
	Code	Source	References
Experience from a wide spectrum of crises. Uncertainty regarding what can happen and how to prepare. Resilience and preparedness work is a challenge. Collaboration and cooperation are crucial. Differences in local crisis training and education	Crisis situations are the known unknown	12	129
	Procedures are needed but relations are key	12	92
	Crisis exercise and training	12	109

Step 3: Common themes	Subthemes
1. *Facing the unknown*	1a. *Risk and emotions* 1b. *Balancing Daily Responsibilities*
2. *Procedures are needed but relations are key*	2a. *Depending on Collaboration* 2b. *Good Crisis Leadership is Knowing Each Other*
3. *We need to train*	3a. *How Often and What Should We Train?* 3b. *The True Value of Training*

*Note:* The column “Code” is identified meaning units; “Source” shows the number of participants (out of the *N* = 12) talking about the code; and “References” shows the number of quotes related to each code.

Finally, in the fourth step, the content of each code group was synthesized and summarized. These analytical texts served to elaborate and detail the nuances associated with each condensate. The analytical texts were validated by comparing the codes and subgroups with the original transcribed interviews. Table 3 gives an overview of the analytical process, and the fourth step is presented in the results section.

### 3.6 Ethical Considerations

The study protocol was approved by the Norwegian Centre of Research Data (NSD/Sikt, ref. code: REDACTED). The participants gave their verbal and/or written consent and received information about their right to withdraw from the study at any time without any following consequences. Established guidelines were observed to protect the anonymity of the participants and to ensure safe storage and use of the data.

## 4 Results

**FIGURE 2:**
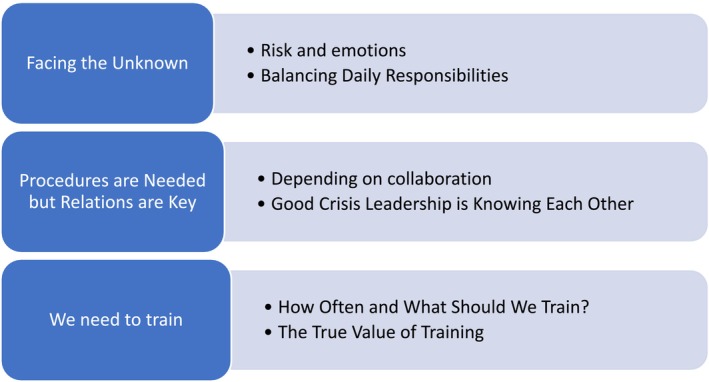
Overview of themes and subthemes from the interviews.

The analysis identified three themes with associated subthemes, describing municipal leaders' experiences with crisis leadership and preparedness in their municipality: (a) *Facing the unknown*; (b) *Procedures are needed but relations are key*; and (c) *We need to train*. Each of these categories will be further elaborated and nuanced with illustrating quotes from the interviews of the municipality leaders (ML) (Figure 2).

### 4.1 Facing the Unknown

#### 4.1.1 Risk and Emotions

The municipal leaders expressed a shared obligation to keep their citizens safe and to bring the local community “back to normal” if a critical incident should take place. The leaders had experienced a variety of crisis situations, such as extreme weather, avalanches, landslides, floods, or forest fires. Events like these had led to adverse consequences such as loss of power, damage to critical infrastructure, material damage, injuries, and death of citizens. Other experiences were extensive fires in public or private properties, large‐scale traffic crashes, loss of life due to incidents at sea or in the mountains, water pollution, and explosions in industrial areas. The pandemic was frequently mentioned as a distinctive and different type of crisis because of its far‐reaching and long‐lasting consequences. One of the leaders stated that the pandemic was *unlike anything we have experienced*, (ML 6). Adding to their personal experiences, the leaders also acknowledged several future risks. Based on their own risk assessment and from witnessing other municipalities, the risk of large cyber‐attacks or weather‐related events was frequently mentioned, as well as nuclear incidents or terror attacks.

This wide range of incidents described by the municipal leaders differed in range, scope, duration, and consequences. One of the leaders said: *there is not one crisis like another*; (ML 7). The leaders acknowledged that future crises could be fundamentally different from what they had experienced before. They expressed an enhanced feeling of uncertainty regarding *what* could happen, *when* it could occur, and *how extensive* the consequences from an event would be for the local community. Like one of the leaders said: *We were told last weekend that we might have to move people [out of an area] over the weekend, right? And then nothing happened. But all the time you are thinking about it. There's a burden in that, carrying that responsibility…. And not quite… Not quite having time off ever, in a way*. (ML 12). This feeling of uncertainty gave rise to emotional distress and the need to be vigilant: *As a municipal director you never have time off, especially when the weather is bad, right?* (ML 4).

Their role and responsibilities in case of a crisis were frequently emphasized by the municipal leaders. The risk‐ and vulnerability assessment, the ongoing war in Ukraine, the refugee situation, and a perceived higher frequency and intensity of extreme weather had led them to acknowledge the need for disaster preparedness.

Still, some of the leaders experienced that disaster preparedness and risk assessment were not given sufficient priority. One of the leaders expressed worry in that *it is the part concerning preventive preparedness and training [that] holds the greatest risk and that we haven't taken a lot of things into consideration* (ML 1). Several leaders detailed their worry over the seemingly mismatch between the identified risks and the local action taken to minimize risks or prepare for possible incidents. Several indicated that crisis preparedness was simply not'on the agend' and that risk awareness was low in the municipality. Some expressed a feeling of unease of not being sufficiently prepared and equipped to face the myriads of potential incidents that could happen. One leader explained: *we are not prepared for, let's say, if someone managed to hack our systems to destroy our servers, for example, then we wouldn't have sufficient back‐up solutions. And we are fully aware of that* (ML 6). The leaders expressed a need and a sense of urgency for more extensive and clearer guidance on advice, priorities, and training in local crisis management. Like one of them said; *When the crisis is there, then it is too late. And that I do not think many people understand* (ML 10).

#### 4.1.2 Balancing Daily Responsibilities

Despite a perceived need to emphasize crisis preparedness, the leaders experienced several obstacles. Given the uncertainty and low probability of crisis situations, it was difficult to maintain sufficient priority on preparedness and training. Setting aside daily work to prioritize crisis preparedness and training, with limited ability to predict the immediate impact and potential long‐term consequences, was considered a tough priority. Compared to the many daily responsibilities, it was difficult to prepare for a remote and unknown future:

You are supposed to provide services in a lot of different areas‐ school, healthcare, and everything‐ the municipalities have enough on their plate with their daily operations, if they are not also to start worrying about and prepare for crises. (ML 10)

Limited resources were frequently mentioned as the main reason for not attending to disaster preparedness, like one of the leaders said: *We know that we are vulnerable in several areas, but we do not have the resources to put people to work to close these gaps* (ML 2). Moreover, the leaders from smaller municipalities understood they had fewer resources compared to larger municipalities. Still, leaders from both smaller and larger municipalities had experienced that local crisis preparedness was easy to “put asid” for the benefit of pressing day‐to‐day responsibilities in the municipality. One of them expressed:

It is hard to get acceptance for spending [dedicated] resources on crisis preparedness when it is seldom ‘in use’. I would like to put more resources in our local security work. But it becomes something that we attend to ‘on the side’, sort of. (ML 12)

In addition to a lack of resources, *perceived risk*, and urgency to prepare for crises were described as a crucial element in local disaster risk preparedness. As large crises that involve the whole municipality are rare, some of the leaders expressed a concern that complacency and low‐risk awareness in the municipality could result in less priority on local emergency preparedness. On the other hand, several leaders explained that real‐life experiences from crisis situations had been instrumental to them in initiating preventive measures or improving crisis management. One of the leaders stated that *we used a lot of time after the [landslide accident] cleaning up what we felt was not working, such as the lines of command or instructions that we felt got messy* (ML 4).

If the local crisis preparedness work were to exceed basic statutory demands, this was often the result of personal initiative. There were great variations in how frequently the municipal leadership had crisis preparedness on the agenda, for example, by testing equipment, carrying out training exercises, or reviewing emergency plans and procedures. The regional authorities appeared to have a vital role in prompting municipal priorities and attention to crisis preparedness, for example, by conducting local audits or organizing joint crisis training. One respondent elaborated on the value of conducting regional exercises involving several municipalities supervised by the county governor:

You get an even better exercise if, for example, the county governor is responsible for the exercise and in a way tests the municipality a bit. I think thats a better way to practice. Rather than just sitting and doing it yourself. […] I know that it is resource‐intensive for the county governor to arrange such things for all municipalities, but [the exercise] gets better when someone from outside comes in, and we who sit in the municipality don't know what is going to happen, right? (ML 6)

Another element that seemed to impact local preparedness was formal knowledge and competence in crisis management and preparedness of the members of the crisis response team in the municipality. The leaders pointed out that, compared to the emergency services, the local crisis response team in the municipality was not selected and trained to manage critical situations.

I don't think there's any requirement in any kind of regulations, at least that I'm aware of, that everyone involved in municipal crisis management must have training or expertise in this area. It's more the expertise you have as a municipal leader that goes into crisis management then, right. (ML 9)

The combination of the lack of resources, perceived risk, and the lack of formal competence of the local crisis team members therefore led to ever‐pressing daily work being prioritized over preparing for potential future incidents:

It is our daily work that is given the highest priority whenever tough priorities must be made, and then local security will be ‘second hand work’, kind of. Even though it should be a part of our core responsibilities, it is probably given the least priority. (ML 11)

### 4.2 Procedures Are Needed but Relations Are Key

#### 4.2.1 Depending on Collaboration

The municipal leaders agreed that risk and vulnerability analysis and updated contingency plans were invaluable tools in providing a sense of security and overview if a crisis should occur. Still, when asked about what they would consider crucial to “good crisis leadership,” they emphasized teamwork and collaboration, both internally in the municipality and with core stakeholders at the local, regional, and national level. The municipal leaders acknowledged that they are fully dependent on close collaboration with many stakeholders. Like one of them said: *I think collaboration is the key word. Collaborating with both locals and emergency services and volunteers, that is sort of the municipality's main responsibility in case of crises. To be a facilitator for the outside world* (ML 9).

The leaders emphasized their responsibility to inform their local citizens and society at large when a crisis emerged. Media was therefore considered important to provide information and mobilize resources. One leader explained why communication and collaboration were so important [*…*]*because there is little you can solve on your own. So, I believe that [relations to other stakeholders] are crucial* (ML 7).

#### 4.2.2 Good Crisis Leadership Is Knowing Each Other

Given their dependence on collaboration and the need to elicit resources from outside the municipality, it was important to maintain good working relations with external stakeholders. A two‐way flow of information was imperative to maintain a shared situational awareness and ensure that the crisis would be managed in the best way possible. Collaborative meetings where roles and responsibilities had been clarified in advance had been invaluable to ensure efficient collaboration when the crisis occurred. The leaders had also experienced barriers and challenges to teamwork and collaboration. For example, when the local crisis management team received little information from the managers on‐site, or when someone expected the municipality to provide services that they could not do, or in situations where stakeholders did not follow up on their responsibilities. Getting to know core stakeholders was seen as important to ensure local crisis preparedness: *The better we know each other, the easier it gets* (ML 4).

Managing different incidents and crises in the past had provided opportunities to know different roles, resources, and procedures, as well as individual differences in risk perception and reactions. One of the leaders explained, *you get to know each other through several incidents you have solved together, so that you possess a kind of unconscious knowledge and competence about what you can expect from the other team members* (ML 1).

Municipality size was perceived to be important in crisis collaboration. Several of the leaders had experienced that it was easier to establish effective working relations with key resources in smaller municipalities. In small municipalities, it was easier to have an overview of the total organization and key persons that could assist in a crisis. One of the leaders from a small municipality shared the following story:

We've been here for a while, the sheriff and me. We have interacted in diverse types of cases, and we know each other well. I will text him if there is anything and he replies, that is where we are at, you know. That is kind of the municipality strength, we are close to people. We are there, there is no Emergency Medical Centre or Police Command Centre. We are close and can solve things quite quickly in communicating [directly] with decision makers. (ML 9)

Several leaders expressed a need for more joint meetings and training with different stakeholders, for example, fire service, police, NGOs, and neighboring municipalities. This would provide a sense of community and an assurance that others would contribute and assist in times of need. One of the leaders compared the neighboring municipalities to a large family:

It's like, a family concerned with helping each other in times of struggle, and several crises has shown just that. When things get tough, we stand up for each other. We are willing to help, no matter where it is. We provide staff if there is an accident other places, right? ‐ and we receive help in return when something happens here. So, I think that is important to bring forward, that without this close collaboration I think many municipalities would be struggling, us included. (ML 4)

### 4.3 We Need to Train

#### 4.3.1 How Often and What Should We Train?

The municipal leaders had different experiences from planning and executing crisis preparedness training. Some checked equipment (i.e., testing radio, satellite phones) or conducted their own tabletop exercises on a quite regular basis. Other municipalities had participated in joint crisis management training with local industry. Still, the majority described that crisis management training was quite infrequent and not according to a predetermined plan. Crisis management training appeared to happen whenever *omeone finds out that we should train* (ML 8).

All the municipal leaders expressed a need to prioritize more training. They also expressed a desire to enhance the *quality* of training. Some had experienced that small‐scale training in the form of well‐planned tabletop exercises could provide valuable insights into roles, responsibilities, and potential pitfalls during crisis management. Well executed, small‐scale exercises could raise awareness and force changes to avoid later problems in a real‐life situation; *you realize that this must never happen again* (ML 7). Larger exercises with multiple trigger events, several key stakeholders, and close‐to‐reality scenarios were seen as most useful. The best training sessions created a sense of realism and urgency that raised pulse rates and induced an emotional reaction in the participants. One of the leaders recently took part in a forest fire exercise and shared:

I believe that, for me and all the others in the local emergency team, and for the mayor and all the other stakeholders, this was a good rehearsal. What we did not realize at the time was that we soon after were to experience an actual forest fire in our municipality. (ML 6)

One of the leaders expressed concern that they needed to train on more diverse types of crisis scenarios, pointing to the fact that a flood and a cyber‐attack would require quite different countermeasures. The leaders were not only concerned about how *ofte*; but also emphasized the need to improve *how* they trained. A shared belief among the leaders was that more frequent training on diverse, but realistic scenarios would be an effective way to develop crisis preparedness. One of the leaders summarized it like this:

We need to train more. That is kind of our number one area of improvement, I think. And to train for different scenarios or different types of crises. It is easy to train for a school shooting or a violent incident, but there is an unbelievable number of crises that can hit a local community. Therefore, getting better at spreading [the training] out on different types of incidents; that is something that I believe we and other municipalities need to be better at. (ML 9)

Although they acknowledged the need for more frequent, high‐quality training, this was largely dependent upon time and resources; like one of them said: *the biggest challenge is to find time in our daily work* (ML 12).

#### 4.3.2 The True Value of Training

Given the uncertainty and complexity associated with disasters, the municipal leaders grappled with the issue of how they could train and prepare for something perceived to be unknown and unpredictabl. Several of the local leaders expressed awareness of the limitations in being prepared for all sorts of crises, given the span of possible incidents. Despite having developed a detailed local risk assessment, it was a significant challenge to detect future crises. The COVID‐19 pandemic was frequently mentioned by the leaders as an example of a crisis that the municipalities were far from prepared to manage. Another example that was mentioned was the sudden and catastrophic landslide killing 11 people in 2020 in Gjerdrum municipality in Eastern Norway. Preparing for such major situations was considered a daunting and nearly impossible task for a municipality, like one leader said: *Being in these situations and not at all knowing what's hitting you, it is impossible to be prepared for that, really* (ML 7).

Still, different forms of training had been a valuable tool for developing trust, understanding, and common goals between team members and different stakeholders. Some of the leaders explained that the greatest value of training is to experience the value of cooperation, communication, information sharing, and the opportunity to establish important relationships during an exercise. Training also provided an opportunity to gain experience from crisis management and collaboration in “peaceful” times. Such collaborative experiences contributed to a sense of safety by *just knowing that there is someone there and that they are available if we need them* (ML 4). Improving interpersonal relations and *teamwork* seemed more important for the municipal leaders than training specific procedures. Training designed to improve collaboration and communication was valued across different crises and incidents, like one of them said: *The exercise itself is not important when it comes to a crisis; it is the fact that we know one another (…) it is the cooperation that is important for when it really goes wrong (ML 10)*.

## 5 Discussion

As societies become increasingly dependent on complex, interconnected systems, they also face greater vulnerability to systemic failures and disruptions (Perrow 1999). This study identifies three key themes in disaster risk governance across 11 municipalities in Western Norway. First, municipal leaders' perceptions of risk and responsibility are shaped by the ongoing challenge of balancing daily operations with crisis preparedness. Second, collaboration, trust, and strong interpersonal relationships with key stakeholders are seen as essential. Third, there is a shared recognition of the importance of training and the need to develop resilient strategies to manage the unexpected. The following sections explore each of these themes in greater detail.

### 5.1 Assessing Risks and Grappling With Uncertainty

Consistent with our expectations, many municipal leaders expressed a strong sense that risks were real and that anything could happen. This pervasive uncertainty made it challenging for them to anticipate or predict the next crisis. Although updated risk analyses had been developed in accordance with government requirements, these documents were often of limited practical value in assessing the actual state of local disaster preparedness.

A notable observation was that real‐life crisis experiences and individual initiative played a significant role in shaping preparedness across many municipalities. The belief that “anything can happen” and the emphasis on maintaining a general state of readiness can be seen as a pragmatic and informed approach to risk governance. Following from this, a way forward could be to develop training programs that enhance the flexibility of emergency response by focusing on common points of failure across both known and unknown hazards (Pescaroli et al. 2023). Addressing systemic risk requires the development of practices that enable the effective use of available resources independently as crises evolve (Linkov and Trump 2019).

In the face of uncertainty and risk, maintaining a general state of readiness may prove more effective than preparing for specific threats that may never materialize. This approach acknowledges that disruptions are likely to occur under unpredictable conditions and avoids the pitfall of overly narrow preparedness strategies (Pescaroli and Alexander 2018). Moreover, such broad‐based preparedness can offer additional societal benefits to address daily issues beyond crisis response (Twigg 2009; Blaikie et al. 2014).

Situations such as the COVID‐19 pandemic, extreme weather, cybercrime, terrorism, and Russia's full‐scale invasion of Ukraine created a heightened awareness of societal safety in Norway (NOU 2023:17 2023). Unsurprisingly, municipal leaders acknowledged these risks and voiced concerns about the potential for future disruptions. They appeared to recognize that their perception of the growing complexity of critical infrastructure could give rise to their perceptions of novel and unprecedented challenges—ones for which existing plans and preparedness frameworks may prove insufficient (Heino et al. 2021). Interestingly, procedures and lessons learned from past experiences could themselves become problematic if future crises differ significantly from those previously encountered. In such cases, relying too heavily on past solutions may hinder rather than help effective crisis response (Heino et al. 2021).

A notable finding from this study is that nearly all municipal leaders reported difficulties in balancing daily responsibilities while also prioritizing crisis preparedness. Some managers highlighted a low level of risk awareness within their municipalities, which negatively impacted local emergency readiness. These challenges reflect broader trends, as the uncertainties and complexities associated with climate‐related risks and interconnected crises increasingly influence both global and local policy priorities (Matejova and Shesterinina 2024).

Municipal directors often have limited influence over political priorities set by governments, placing them in a difficult position—caught between political agendas and their responsibilities to safeguard the public and ensure sustainable service delivery. This tension underscores the importance of informing the public about risks and involving citizens in preparedness planning, which is a critical step toward building a more resilient society (Lidskog and Rabe 2022). Public engagement is essential for resilience, as it fosters shared responsibility and enhances the legitimacy of preparedness efforts. Raising public awareness can also help shift local priorities and secure greater funding for safety measures, which often compete with frontline service provision in municipal budgets (McConnell and Drennan 2006).

Effective contingency planning requires a structured approach to identifying and prioritizing future threats (McConnell & Drennan 2006)—a task that is inherently challenging. The high degree of uncertainty and the low frequency of some crises help explain why municipal leaders often struggle to develop specific plans for potential incidents (Enander et al. 2015). This may also account for the consistently low prioritization of crisis preparedness observed in previous studies (Eriksson 2023), as well as in the current study.

One promising strategy is to allocate resources toward addressing common points of failure shared across various low‐probability, high‐impact scenarios. This approach can support the development of more effective and adaptable preparedness measures (Pescaroli et al. 2023).

### 5.2 Interpersonal Relations, Collaboration, and Collective Values Are Key to Local Crisis Preparedness

Municipal leaders rely heavily on collaboration and external support to manage crisis situations—an understandable necessity given the small population size of many Norwegian municipalities. They emphasized the importance of teamwork within the municipality and the need to maintain strong working relationships with stakeholders at the local, regional, and national levels.

Although the value of risk assessments and contingency plans was acknowledged, personal relationships were often regarded as even more critical. This finding aligns with previous research suggesting that effective crisis preparedness extends beyond formal plans and procedures—it is fundamentally about people (Hede 2017; Eriksson 2023).

Municipal leaders consistently emphasize the importance of interpersonal relationships and their need to mobilize local resources based on personal knowledge. Several explicitly stated that effective crisis leadership hinges on trust and strong personal connections. They also recognized that teamwork can be undermined by conflict, aggression, or competing priorities (Eid, Hansen, et al. 2023).

Establishing trust in local leadership and investing in collaborative efforts were therefore seen as essential components of crisis preparedness (Hyllengren et al. 2011). A notable finding from this study is the emphasis placed on psychosocial factors—such as interpersonal relationships, trust, and team dynamics. These elements, often associated with collectivist cultures (Taarup‐Esbensen 2022), were viewed by municipal leaders as critical to risk governance and crisis preparedness. The findings suggest that these psychosocial dimensions should be more explicitly explored and integrated into disaster preparedness training.

### 5.3 How to Prepare and Train for the Unknown?

Norwegian municipalities are legally required to ensure crisis preparedness and training; however, these requirements are broadly defined and subject to local interpretation and prioritization. Ideally, resilience training should equip professionals with the situated capability to withstand and effectively respond to critical situations in their specific work contexts (Ketelaars et al. 2024). Stress‐testing the resilience of a system—such as a municipality—can enhance understanding of its structures and dynamics, while also informing more strategic resource allocation (Linkov et al. 2022; Twigg 2009; see also 't Hart et al. 1993).

The current study revealed significant variation in both the frequency and content of crisis preparedness training across municipalities. Several had participated in annual regional training sessions organized by regional authorities. These sessions were generally valued for providing updated information on procedures, legislation, and national requirements, such as conducting risk analyses and complying with regulatory frameworks. However, the focus of these sessions was largely on static knowledge, with limited attention to process‐oriented learning, interpersonal dynamics, and the more fluid aspects of crisis management.

Few municipalities had initiated their own crisis management training, highlighting a gap between formal expectations and the realities of local implementation. This suggests a need for more dynamic, context‐sensitive training approaches that emphasize collaboration, adaptability, and psychosocial competencies in addition to technical knowledge.

Despite limited resources, leaders from smaller municipalities generally considered themselves well prepared to handle a crisis. Their close relationships with key stakeholders and deep knowledge of local social networks were seen as major assets. However, all municipal leaders expressed a clear desire and need for more frequent training.

The true value of crisis training, as perceived by the leaders, lay in its ability to build trust and strengthen interpersonal relationships through shared exercises. Yet, such training was rarely initiated or conducted locally; highlighting a gap between perceived preparedness and the actual implementation of structured crisis management practices.

A constructive way forward is to encourage municipalities to develop competencies in process‐oriented training. This type of training offers opportunities to critically reflect on past practices and prepare crisis management teams to devise alternative solutions when faced with unfamiliar challenges (Heino et al. 2021). To be effective, such training should involve both administrative and political leaders, fostering a shared and realistic understanding of local disaster risk governance strategies.

Simulation‐based training can further enhance preparedness by helping key decision‐makers understand the interdependencies between their roles and those of co‐decision‐makers, and how these relationships influence overall performance and crisis response (Wehrle et al. 2022). Looking ahead, integrating virtual reality (VR) or mixed reality (MR) technologies could simulate collaboration and interaction within and between municipalities (Altan et al. 2022). These immersive environments may help leaders adapt to surprise and uncertainty by gradually building their capacity to respond across varying levels of complexity (Streets and Glantz 2000; Alderson et al. 2022).

Training formats ranging from technology‐based gaming sessions to traditional tabletop exercises can promote best practices in stress testing, enhance understanding of common failure points, guide resource allocation, and strengthen cross‐agency coordination (Linkov et al. 2022).

### 5.4 Limitations and Future Research

This exploratory study, based on interviews with 12 municipal leaders from variously sized Norwegian municipalities, offers insights into local disaster risk governance. Although the small sample limits generalizability, the findings highlight key challenges and opportunities. Future research could expand through comparative studies of political and managerial leaders, larger‐scale quantitative surveys, or in‐depth case studies. There is also potential in exploring training innovations—such as simulations and virtual reality—to enhance collaboration and crisis response. International comparisons using the same methodology could further illuminate the role of cultural and organizational factors in disaster preparedness.

### 5.5 Conclusions

This study offers a significant and unique contribution to studies of crisis preparedness and systemic risk in Norway, focusing on insights from municipal leaders. A key point from the results is recognizing the uncertainty and concern experienced by many municipal managers and the emotional stress associated with preparing for low probability, high‐impact incidents. A notable finding is the appreciation and significance of interpersonal relations, local expertise, trust, and knowledge within and outside of the municipality. Clear understanding was evident that effective teamwork may suffer from conflict, aggression, or conflicting priorities. Advice on preparing and training for the unknown and for improved resilience was also prominent, while raising practical difficulties associated with prioritizing crisis preparedness training and attending to pressing day‐to‐day obligations. In fact, few of the municipalities had conducted crisis management training from their own initiative, indicating a gap between formal expectations and local reality.

These experiences ought to be applied around Norway, and they are likely transferable to many other places, to improve crisis preparedness and dealing with systemic risk, especially through facing the unknown, ensuring good interpersonal relations, and improved training. Aside from transferability, further research should involve a more rigorous survey aimed at disaster risk governance in rural municipalities and systematic studies of process‐training and digital gaming exercises, all of which would further learn from and support municipal leaders.

## Author Contributions

S.S. and J.E.: conceptualization, methodology, data analysis, writing and editing. G.P. and I.K.: writing – reviewing and editing.

## Conflicts of Interest

The authors declare no conflicts of interest. Jarle Eid did not take part in the editorial process involving this study.

## Data Availability

The data that support the findings of this study are available on request from the corresponding author. The data are not publicly available due to privacy or ethical restrictions.
